# Functional fingerprinting for the developing brain using deep metric learning

**DOI:** 10.1162/IMAG.a.1112

**Published:** 2026-01-27

**Authors:** Rui Xu, Shuwan Zhao, Zhengyi Liu, Qianhui Jin, Zeyao Wang, Dongxu Liu, Kun Zhao, Suyu Zhong, Jiaying Zhang, Yong Liu, Ting Qi, Yongbin Wei

**Affiliations:** Center for Artificial Intelligence in Medical Imaging, School of Artificial Intelligence, Beijing University of Posts and Telecommunications, Beijing, China; Queen Mary School Hainan, Beijing University of Posts and Telecommunications, Hainan, China

**Keywords:** brain fingerprinting, individual variability, brain development, fMRI, individual identification

## Abstract

Brain fingerprinting offers a promising method for delineating the unique functional architecture of individual brains using neuroimaging data. Here, we present a novel deep learning framework—Metric-BolT—for brain fingerprinting and use it to characterize distinct developmental trajectories during childhood and early adolescence. Based on longitudinal neuroimaging data, the extracted brain fingerprints achieved identification accuracies of 97.6% for two data runs acquired within a single session and 86.6% for runs spanning 4 years. Notably, the most discriminative brain fingerprints were driven by higher-order association cortices, particularly regions of the default-mode network. Moreover, we showed these fingerprints to correlate with cognitive abilities, such as fluid and crystallized intelligence, and to exhibit significant genetic associations, with stronger genomic relationships observed among individuals with more similar fingerprint patterns. Genes associated with brain fingerprinting tended to show upregulated expression in the frontal cortex, particularly in late childhood. Together, our study presents an innovative computational approach for brain functional fingerprinting and provides novel insights into individual variability in adolescent neurodevelopment.

## 1 Introduction

Individual differences in both the function and structure of the brain have been widely observed in adults (Finn & Todd Constable, 2016; Kanai & Rees, 2011; Liao et al., 2017; Llera et al., 2019; Seghier & Price, 2018) and are particularly pronounced during development (Foulkes & Blakemore, 2018; Mills et al., 2021). A considerable variation in the functional connectivity (FC) of association regions, in contrast to a relatively limited variation in primary sensorimotor and visual regions, has been noted as early as the third trimester of brain development (Hu et al., 2022; Xu et al., 2019). Similar inter-individual variation patterns of FC have also been reported in later development stages of childhood, with several higher-order brain networks further correlating with variations in children’s cognitive abilities (Marek et al., 2019). Characterizing the unique pattern of human brain development is thus critical for understanding the complex developmental trajectories underlying children’s behavioral and neurocognitive changes.

In light of the individual variability, researchers have introduced the “brain fingerprint”, which refers to distinctive neural signatures that enable the identification of individuals within a population (Finn et al., 2015). Brain fingerprint was proposed as a promising approach to characterize the uniqueness of brain functioning based on functional magnetic resonance imaging (fMRI) data. Using the functional brain connectome, Finn et al. (2015) precisely identified specific individuals from a large cohort of young adults and distinguished different brain activation patterns across tasks. These brain fingerprints rest upon FC matrices and exhibit stable and significant individual differences in FC distributed across three higher-order cognitive networks (i.e., default-mode, dorsal attention, and fronto-parietal networks), reflecting inherent functional dynamics of an individual’s brain connectome (Liu et al., 2018). Moreover, the accuracy of individual identification for young adults, either using brain fingerprints of static or dynamic FC, remains stable over several months (Menon & Krishnamurthy, 2019), suggesting that the intrinsic individual connectivity patterns do not significantly change in adults. Furthermore, brain fingerprints based on the functional connectome have demonstrated the capability of predicting cognitive traits or clinical symptoms in mental conditions (Shen et al., 2017; Supekar et al., 2022). These findings stress that brain functional fingerprinting offers a straightforward and reliable approach for the examination of individual variability.

While brain fingerprints robustly identify the unique individual among adults, it is a different scenario in the context of developing populations, given the dynamic nature of the brain during maturation. Using the functional connectome, lower individual accuracies (below 60%) have been observed when identifying individuals among children and adolescents compared with those of adults (around 65%), indicating that individuals’ functional connectomes continue to become unique during development (Kaufmann et al., 2017). Moreover, longitudinal studies have shown that brain fingerprinting remained valid over periods of 3 months to 2 years in adolescent populations; however, the accuracy of identification significantly dropped to approximately 77% compared with that of adults (usually over 90%) (Horien et al., 2019; Jalbrzikowski et al., 2020). Novel fingerprinting methods are imperative for identifying more refined developmental patterns of brain functioning, specifically functional fingerprints that could effectively differentiate individuals during a longer period of development.

Various methods have been developed for extracting brain fingerprints using fMRI data. Originally, Finn et al. (2015) utilized an FC-based correlation approach, with individuals identified by calculating the correlation between two functional connectomes. Recent advances have incorporated brain dynamics through time-windowed analyses, segmenting time series into different windows to identify when the best identification occurs and at what time scale (Van De Ville et al., 2021). Although these methods have made significant progress in identifying individuals, there are still some challenges. Notably, the identification accuracy using FC decreases significantly as the number of subjects increases, with an estimated accuracy of 62% for a sample size of 10,000 and 42% for 100,000 subjects (Waller et al., 2017). More recently, deep learning methods have further opened up new avenues for brain fingerprinting by maximizing inter-subject variances in embedding space (Chen & Hu, 2018; Hannum et al., 2023; Kim et al., 2021; Sarar et al., 2021; Shojaee et al., 2019; Zhao, 2025). For instance, an effective approach combines autoencoders or conditional variational autoencoders with sparse dictionary learning modules to capture shared information among subjects, while using the residuals with enhanced inter-individual variability as brain fingerprints for individual identification (Cai et al., 2021; Lu et al., 2024). These methods demonstrate great potential in capturing the unique functional fingerprints of individual brain networks, yet more refined methodologies with enhanced identification precision during development as well as more solid neurobiological explainability are needed.

In the current study, we propose a deep learning framework based on distance metric learning for longitudinal identification of brain fingerprints in early adolescence. Conceptually, this method refines fMRI data into a highly optimized neural “fingerprint”, thereby allowing the model to capture the essential features that make an individual unique and remain distinctive over time. Utilizing the blood-oxygen-level-dependent transformer (BolT) (Bedel et al., 2023), this framework optimizes the feature space by maximizing the inter-subject distances while minimizing intra-subject distances. Using longitudinal resting-state fMRI data from the Adolescent Brain Cognitive Development (ABCD) dataset of children aged 9–10 years at baseline, we achieved high identification accuracies of 97.6% within one scan session and 86.6% over a 4-year period. Further, we demonstrated key regions within higher-order cognitive networks with significant individual variability during development and highlighted the associations of brain fingerprints with cognitive abilities and genetic factors.

## 2 Methods

### 2.1 MRI data

MRI data were obtained from the ABCD Study (data release 5.1; DAR ID: 16920), which includes longitudinal MRI from 11,892 9- to 10-year-old children (Auchter et al., 2018; Casey et al., 2018; Garavan et al., 2018; Karcher & Barch, 2021; Uban et al., 2018). These participants and their families were recruited through schools and community settings across 21 sites in the United States, representing a diverse cross-section of the U.S. population in terms of education, race, and socioeconomic status. The ethical review of the study was approved by the University of California’s central Institutional Review Board (IRB) for most sites, while local IRBs approved the study at other locations. The current study used data from 1,325 participants with an initial age range of 9 to 10 years, excluding all subjects without the 4th-year follow-up data and those without either T1 imaging or resting-state fMRI data (Supplementary Fig. S1). The longitudinal data for this final sample span three time points: a baseline session, a 2nd-year follow-up, and a 4th-year follow-up, with two separate resting-state scans acquired at each session. Specifically, 1082 subjects have data from both the baseline and 4th-year sessions, 1000 subjects have data from the 2nd- and 4th-year sessions, while 1227 subjects have data exclusively from the 4th-year session.

### 2.2 Data preprocessing

Minimally processed T1-weighted MRI data and resting-state fMRI data were obtained from the ABCD study. Details of scanning parameters and processing procedures have been reported in Hagler et al. (2019). Minimally processed T1-weighted MRI data were further processed using FreeSurfer (v.6.0) for brain segmentation and cortical mantle reconstruction. The FreeSurfer output was manually inspected by experienced researchers. The reconstructed cortical mantle was parcellated into 219 distinct regions according to a subdivision of the Desikan–Killiany atlas (Cammoun et al., 2012; Desikan et al., 2006).

In parallel, minimally processed resting-state fMRI data were further processed using CATO (v.3.1.2) through the following steps (de Lange et al., 2023). First, resting-state fMRI data were realigned using MCFLIRT to correct for any displacement during participant scanning. Second, data were co-registered with the T1-weighted data to overlap with the resultant cortical parcellation maps under the DK-219 atlas. Then, six motion parameters, first-order drifts of the six motion parameters, and mean signal intensity of voxels in white matter and CSF were regressed through linear models. Bandpass filtering was further conducted to eliminate frequencies outside the 0.01 to 0.1 Hz range, retaining only signals within the desired frequency band. Notably, scrubbing was not applied to ensure the same length of time series across participants. Finally, time series with lengths of 375 time points (i.e., 300 s) per scan run were used in the following analyses.

### 2.3 Metric-BolT model construction

**Fig. 1.**
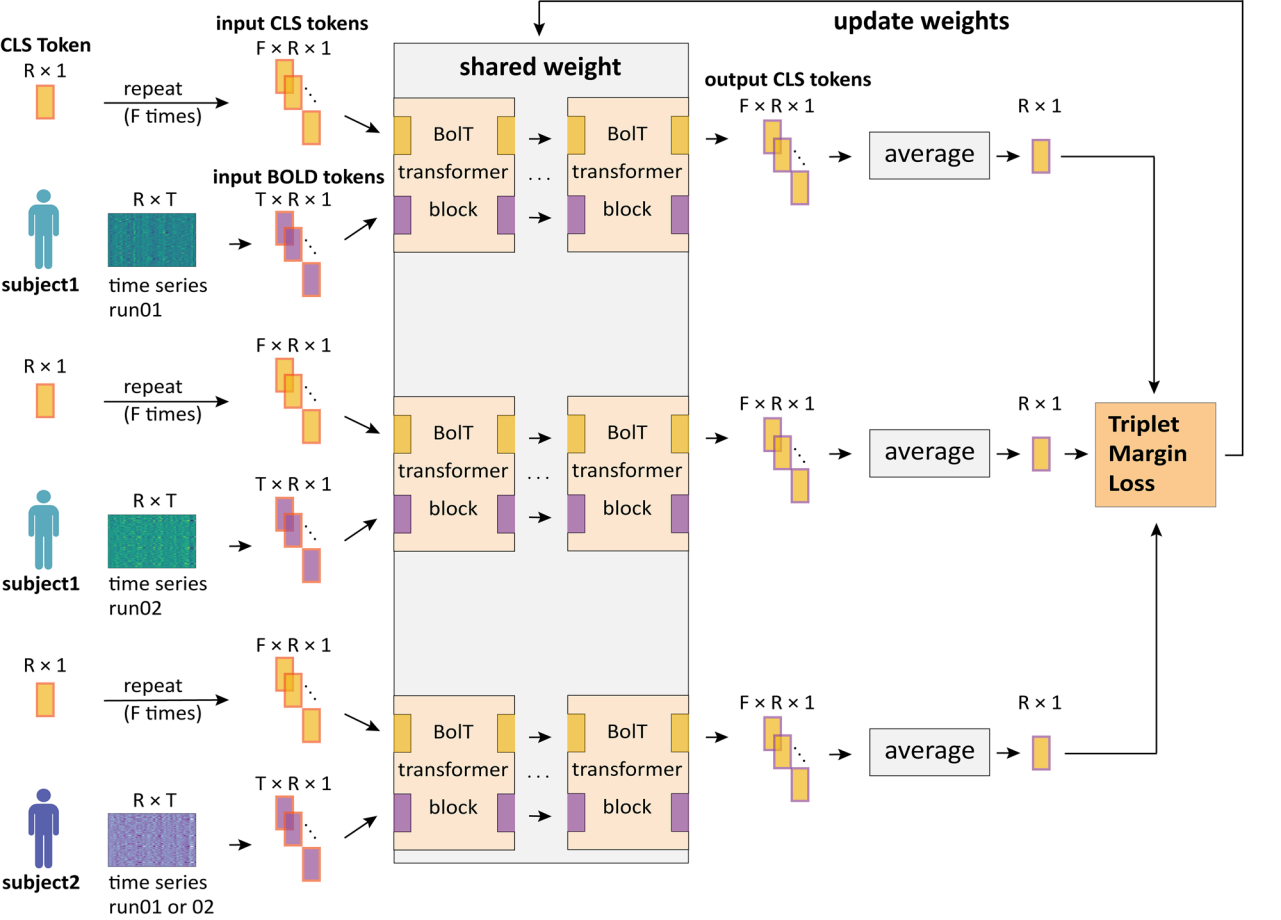
The scheme of Metric-BolT. Each training iteration involves three time series as inputs: two from the same subject (as the anchor and positive sample) and one from a different subject (the negative sample). Each time series matrix is divided into BOLD tokens and then processed through transformer blocks. Within each transformer block, the time series is further divided into multiple temporally overlapping windows, with each window containing multiple BOLD tokens. BolT also initializes a dedicated classification (CLS) token for each time window, which starts as an identical vector and evolves into unique summaries of their local contexts by interacting with BOLD tokens to capture task-relevant features. The output CLS tokens are then averaged to produce the final brain fingerprint (see Supplementary Methods). The model is trained and updated using TripletMarginLoss as optimization criterion. All three samples in a single training iteration share the same model weights. R: number of brain regions; T: time series length; F: number of time windows.

To extract brain fingerprints during brain development, we integrate the BolT model (Bedel et al., 2023) with deep metric learning, further referred to as “Metric-BolT”. This framework extracts brain fingerprints and keeps the brain fingerprints from the same subject more proximate, and those from different subjects more distinct (Fig. 1). Specifically, each participant is treated as a class, and the matrix of fMRI time series of each participant is treated as a sample. The deep learning model then maps each sample (i.e., time series) into a high-dimensional space. In this space, the ideal goal is to achieve a configuration in which the maximum intraclass distance is always smaller than the minimum interclass distance. This means the data from different subjects are well separated, while the data from the same subject are closely clustered together.

This approach consists of two key steps: (1) encoding fMRI time series into feature vectors, referred to as brain fingerprints, and (2) comparing the distance of feature vector pairs. In the first step, we employed the BolT model (Bedel et al., 2023) to optimally represent the fMRI data in the embedding space, resulting in a highly representative encoding of the original fMRI time series (see details in Supplementary Methods). In the second step, we quantified the distance between feature vectors using 1 minus cosine similarity and employed TripletMarginLoss with cosine similarity metric as our loss function (Schroff et al., 2015), which is defined as

L(a,p,n)=max{d(a,p)−d(a,n)+margin,0}, (1)

where L represents the triplet loss function, a, p, and n denote the anchor, positive sample (same class as a), and negative sample (different class from a), respectively.

Specifically, within each training batch, a pair of time series from a single subject was treated as the anchor and positive samples, while time series from all other subjects in that batch served as the negative samples. The margin is a threshold used to control the distance between positive and negative samples. When d(a,p)−d(a,n)+margin>0 , indicating the negative sample is not sufficiently far from the anchor compared with the positive sample, this difference is recorded as the loss. Otherwise, the loss is set to 0. This mechanism encourages the model to learn that the distance between anchor-positive pairs is smaller than anchor-negative pairs by at least the margin, thus pushing similar samples closer and dissimilar samples farther apart, as illustrated in Figure 1. This two-step process allows us to establish brain fingerprints for each individual and effectively distinguish between different individuals in the learned embedding space.

To evaluate our model’s performance and robustness, we employed a standard 80%–10%–10% hold-out split and a rigorous site-conserved nested 5-fold cross-validation (Supplementary Methods). The latter approach specifically ensures that all participants from the same site—including related individuals (twins/siblings) who share genetic and environmental factors—are kept together in the same fold. This provides a more rigorous estimate of generalization by accounting for site and family effects. We also compared our model’s performance against two other prominent models (Cai et al., 2021; Lu et al., 2024) to demonstrate its effectiveness.

In all experiments, fundamental parameters determined by hardware constraints or data length (e.g., batch size = 8), as well as standard configurations inherited from the original BolT model, were held constant. Meanwhile, our hyperparameter tuning focused on one specific variable (i.e., margin) and was aligned with each validation strategy. For the 80%–10%–10% split, we performed manual tuning on the 10% validation set; the margin was tested with values [0.3, 0.5, 0.7, 0.8, 0.9, 1.0], with 0.7 selected as the optimal setting. For the site-conserved five-fold cross-validation, we adopted a nested cross-validation framework. Within each outer iteration, Optuna was utilized to perform an inner cross-validation on the corresponding training data to automatically identify the optimal hyperparameters specific to that fold (Akiba et al., 2019). The fold-specific optimal hyperparameters and the complete model configuration are detailed in Supplementary Tables S1 and S2, respectively. Additionally, given that the site-conserved partitioning results in varying fold sizes, the specific training and testing sample counts are provided in Supplementary Table S3.

### 2.4 Individual identification

**Fig. 2.**
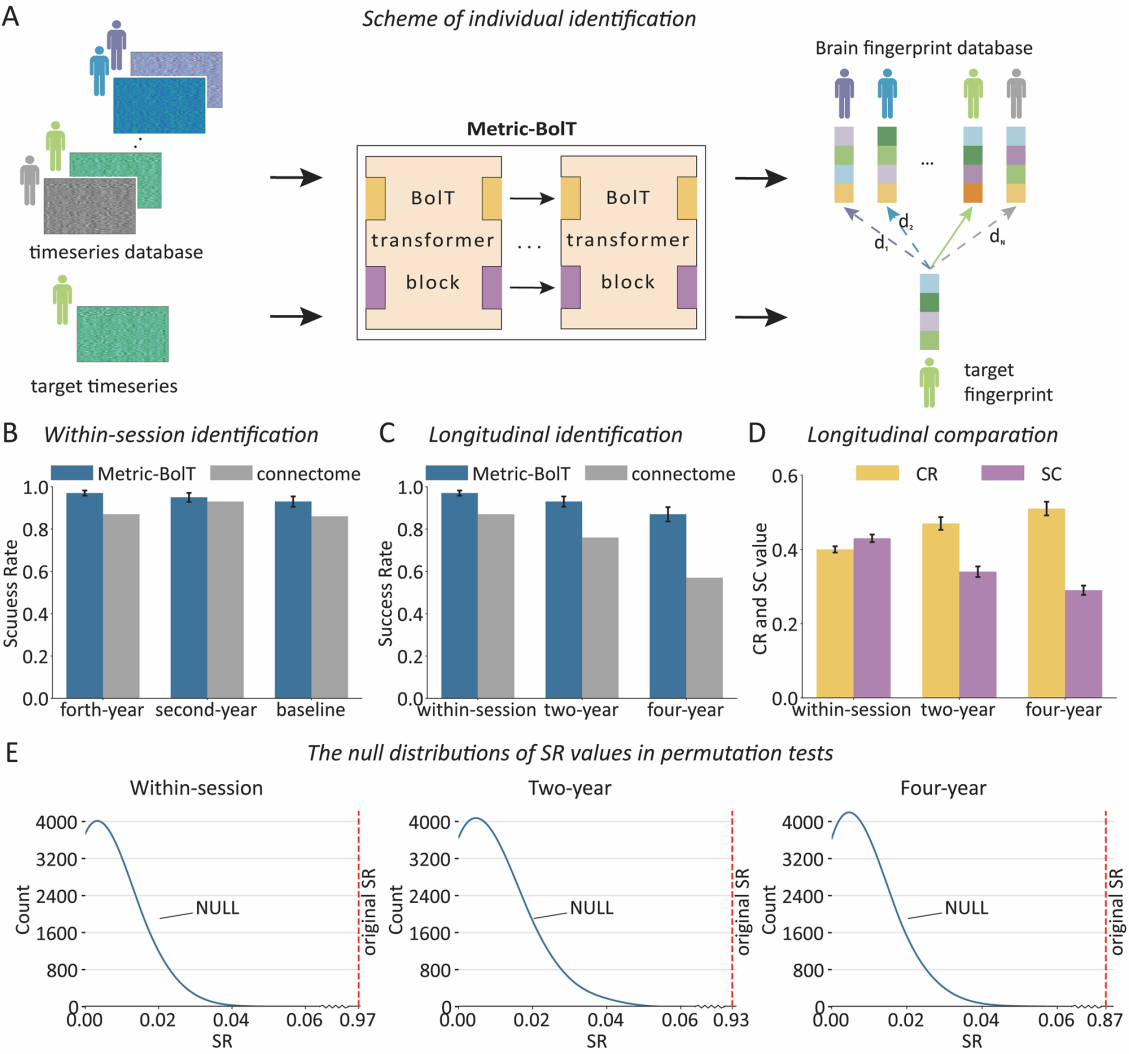
Precision in individual identification. (A) Individual identification scheme. Time series are transformed to brain fingerprints using Metric-BolT. Given a target data sample, distances are computed between the target brain fingerprint and each brain fingerprint in the brain fingerprint database, identifying the fingerprint with the minimum distance. (B) SR of individual identification using Metric-BolT and connectome-based approach at baseline, 2nd-year follow-up, and 4th-year follow-up data. Error bars represent the standard deviation (SD) across 15 model checkpoints evaluated on the test set. The multiple success rates were generated by evaluating distinct model checkpoints saved at various stages of a single training run. (C) SR of individual identification using Metric-BolT and connectome-based approach for within-session (two runs from 4th-year follow-up), 2-year (runs from 4th- and 2nd-year follow-up), and data with a 4-year interval. Error bars represent the standard deviation (SD). (D) The ratio of average intra-class to inter-class distances (CR) and the silhouette coefficients (SC) for different time intervals. Error bars represent SD. (E) The null distribution of SR values in 10,000 permutations. The solid blue line indicates the actual distribution of SR, created using spline interpolation for visualization purposes, while the red dashed line represents the original SR.

We applied Metric-BolT to resting-state fMRI data from the ABCD dataset, including fMRI time series data of individuals scanned at three time points (i.e., baseline, 2, and 4 years later). To validate the effectiveness of the trained model, specifically the uniqueness and stability of brain fingerprints, we conducted individual identification experiments across six cases: two runs from three within-session and three cross-session longitudinal cases on the test set. Prior to the identification process, all time series from both runs were processed through Metric-BolT to generate brain fingerprints for each subject. Then the feature vectors (i.e., brain fingerprints) from one run were used to create the brain fingerprint database, $D=[X_{i},i=1,...,N]$ , where i stands for the participant, N is the number of subjects in the test set. Feature vectors from the other run were used as the target for identification. In an iterative process, the identity of a target feature vector $Y_{i}$ was predicted by comparing it with all vectors in D. The predicted identity was determined by finding the vector in D with the minimal distance to $Y_{i}$ (Fig. 2A). Note that we performed identification with replacement, meaning each target feature vector could be matched to any subject in the database, regardless of previous matches. This iterative process was repeated for all feature vectors in the target run. To ensure robust evaluation, we employed a bi-directional testing strategy: we swapped the target and database runs and repeated the entire identification procedure.

Three key metrics were established to evaluate the efficacy of our experimental approach: identification success rate (SR), averaged intra- and inter-class distance ratio (CR), and the Silhouette coefficient (SC) (see details in Supplementary Methods). To assess the statistical significance of the identification accuracy, we used a nonparametric permutation test. This approach involved 10,000 independent tests, where in each test, subject identities in the target set were randomly permuted. After each randomization, the identification algorithm was applied, and the SR was recorded. This process generated a null distribution of SR scores. By comparing the original SR with this null distribution, we determined the statistical significance of the results, evaluating the method’s performance beyond the chance level.

To rigorously assess the true generalization ability of our Metric-BolT method, we designed a zero-shot transfer experiment. The model was first pre-trained on the ABCD dataset to identify individuals across three distinct temporal conditions: within-session, 2-year, and 4-year intervals. Subsequently, without any fine-tuning or further training, this pre-trained model was directly applied to perform individual identification tasks on the Human Connectome Project (HCP) (Van Essen et al., 2013) dataset. The HCP test set consists of resting-state fMRI (rs-fMRI) scans from 893 adult participants (age range: 23–34 years), each with multiple scanning sessions following standardized HCP protocols. For the identification task, the model utilized two runs within a scan session, with a primary time series length of 1200 (864 s). The performance was benchmarked against the connectome-based approach, and an additional analysis was conducted by adjusting the fMRI time series length to 375 time points.

### 2.5 Brain fingerprint interpretation

We calculated the contribution of each brain region to gain a deeper understanding of the underlying brain functional dynamics that drive brain fingerprinting. We first used an attention mechanism-based approach to quantify the contribution of each BOLD token to the brain fingerprint, resulting in token-level importance for each participant (Supplementary Methods). Based on this, we used a random forest approach to obtain the importance of brain regions in fingerprinting, translating this token-level importance into brain region importance. By labeling the top five most important BOLD tokens as 1 and the top five least important as 0, we created a binary classification task. The resulting model weights, *W*, offered a quantitative measure of each brain region’s contribution to the brain fingerprint.

The cortical patterns of regional contributions were further annotated in terms of the seven resting-state brain functional networks (Yeo et al., 2011) and distinct biological brain maps. First, using the correspondence between nodes in the DK219 brain atlas and seven resting-state functional networks provided by neuromaps (Markello et al., 2022), we performed *t*-tests comparing each network with all other networks, as well as *t*-tests comparing higher-order cognitive networks with lower-order sensorimotor networks. Separately, distinct biological brain maps were analyzed using the DK219 parcellation. The data were first mapped to the atlas, and Pearson correlations were calculated for each of the 219 brain regions. Subsequently, 1,000 spatial permutations were performed to ensure that the permuted data retained spatial autocorrelation (Alexander-Bloch et al., 2018; Burt et al., 2020), followed by computing *p*-values through permutation tests to assess statistical significance. Finally, FDR correction was applied to control the false discovery rate and address multiple testing issues.

### 2.6 Correlation analysis between brain fingerprints and cognitive abilities

To examine the association between brain fingerprints and cognitive abilities, we employed least squares regression and tested the significance of regression coefficients. Specifically, we selected measures of higher-order cognition, including fluid intelligence (Cognition Fluid Composite, mean ± SD: 96.944 ± 17.685, range: 58–138), crystallized intelligence (Crystallized Composite, mean ± SD: 108.252 ± 20.932, range: 36–165), executive function (Dimensional Change Card Sort, mean ± SD: 98.330 ± 16.578, range: 77–155), and general cognitive ability (Cognition Total Composite, mean ± SD: 102.832 ± 19.269, range: 59–154) as described in prior research (Gray et al., 2020). Given the substantial individual differences in neural development during childhood and adolescence and the non-linear effects of age on cognitive performance, we utilized age-corrected cognitive scores to eliminate potential assessment bias caused by varying developmental rates, thereby more accurately reflecting individual cognitive capabilities. We then constructed a linear regression model with brain fingerprint vectors as independent variables and cognitive test scores as the dependent variable. After fitting the model using the least squares method, we proposed a null hypothesis that all regression coefficients equal zero. We then calculated the F-statistic and its corresponding *p*-value for the regression coefficients to assess whether brain fingerprints could significantly explain the variance in cognitive abilities.

### 2.7 Genetic analysis in terms of brain fingerprints

We examined whether genetic factors affect the extracted brain fingerprints. We used genotype data and computed the Identity by Descent (IBD) measure, represented by the pi_hat statistic. IBD refers to the inheritance of identical genetic material from a common ancestor (Supplementary Methods) (Thompson, 2013), with the genomic relationship quantified by pi_hat. Pi_hat ranges from 0 to 1, with values above 0.5 typically indicating first-degree kinship. We conducted experiments across three time intervals through the following steps: we categorized the subjects into two groups based on their pi_hat values, with pi_hat > 0.5 defining the group exhibiting a strong genomic relationship and pi_hat ≤ 0.5 representing genomically unrelated. We created new training, validation, and test sets using only genomically unrelated participants. Training then proceeded on this newly defined training set to develop a new Metric-BolT. Subsequently, we tested the trained model on pairs of genetically related subjects and pairs of genetically unrelated subjects, comprehensively assessing the genetic specificity of brain fingerprints. The number of subject pairs with close genetic relationships and without such genetic similarities for the within-session, 2-year, and 4-year intervals was 80 and 6,240, 44 and 1,848, and 62 and 3,720, respectively. Finally, we used two-sample t-tests to compare group means of distances. A *p*-value of < 0.05 was considered statistically significant across all tests.

We further identified genes potentially implicated in neural individuality during the extraction of brain fingerprints, given that the expression levels of genes vary substantially across brain regions (Hawrylycz et al., 2015). The AHBA (E. H. Shen et al., 2012), which has already yielded novel insights into adolescent brain development (Kirsch & Chechik, 2016; Whitaker et al., 2016), contains expression data for 15,633 genes, which can be mapped to the DK-219 atlas using abagen (Markello et al., 2021). We first calculated the correlation between regional gene expression and brain regional contribution profiles, then performed 1,000 spatially autocorrelation-preserving permutations using BrainSMASH (Fulcher et al., 2021) to compute *p*-values. Finally, we selected the most positively correlated genes with FDR-corrected *p* < 0.1 (a lenient threshold was used due to the high correlation and dependence among gene expression profiles). The identified genes were then annotated in the context of gene expression and biological pathways using FUMA GWAS.

## 3 Results

### 3.1 Metric-BolT identifies individuals longitudinally

The performance of Metric-BolT was evaluated in identifying individuals across three time points during brain development. Metric-BolT achieved an SR of 93.4% for the two runs of baseline data, 95.2% for the 2nd-year follow-up data, and 97.3% for the 4th-year follow-up data (Fig. 2B). This result confirmed an increasing distinctiveness of the functional fingerprints along with brain development. Moreover, longitudinal identification, taking data from a baseline run as the fingerprint database and testing individuals from a 2nd-year run, showed an SR of 90.9%, while testing the 4-year run showed an SR of 86.6%. Testing the 4th-year individuals against the 2nd-year fingerprint database further revealed an SR of 92.6%.

Reversing the roles of the brain fingerprint database and testing samples revealed similar results: within-session runs showed an SR of 94.0%, 96.3%, and 97.6% at the baseline, 2nd-year follow-up, and 4th-year follow-up, respectively. Swapping the baseline run and 2nd-year run, the 2nd-year run and 4th-year run, and the baseline run and 4th-year run, respectively, showed an SR of 91.6%, 93.0%, and 86.6% (Fig. 2C). Results from the site-conserved nested five-fold cross-validation yielded mean SRs of 97.1% (within-session at the 4th-year follow-up), 90.2% (2-year interval, between the 2nd-year and 4th-year runs), and 86.1% (4-year interval, between the baseline and 4th-year runs) (Supplementary Table S4). These high SRs in individual identification indicate that the identified brain fingerprints are effective and maintain relatively stable over an interval of even 4 years during development.

Additionally, the achieved SR outperforms the SR obtained using the functional-connectome-based approach (Fig. 2B, C), as well as those of two benchmark deep learning methods (see Supplementary Table S5). We also validated our results on a fixed cohort of 819 subjects present across all time points, which yielded mean SRs of 97.6% (within-session at the 4th-year follow-up), 93.9% (between the 2nd-year and 4th-year runs), and 85.4% (between the baseline and 4th-year runs).

In addition to SR, we used CR and SC to evaluate the individual identification performance. CR rose from 0.41 for within-session individual identification to 0.47 for identification with a 2-year interval, and to 0.5 for identification with a 4-year interval (Fig. 2D). Meanwhile, SC dropped from 0.45 to 0.34 and then to 0.30 along with the time intervals (Fig. 2D), also suggesting that a higher individual identification performance is associated with a shorter time interval.

The permutation tests revealed that the maximum SRs were 6.50% for identifications within one session, 6.00% for the 2-year interval, and 6.42% for the 4-year interval, obtained under random permutations (all *p* < 0.001, 10,000 permutations) (Fig. 2E). These results confirmed the achieved SRs to be higher than what can be expected by chance.

### 3.2 Brain fingerprint is driven by association regions in the brain

**Fig. 3.**
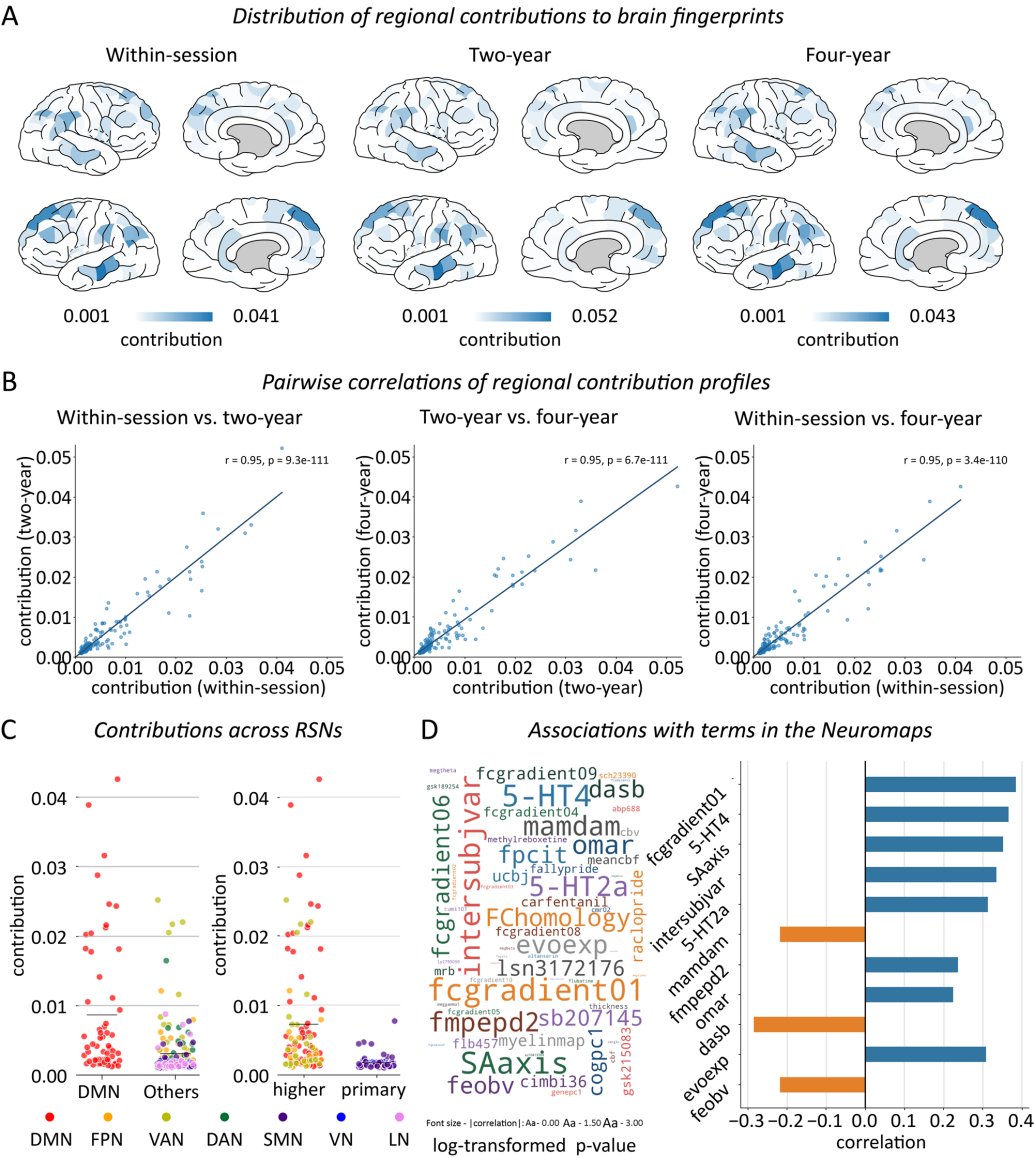
Regional contributions to the extracted brain fingerprints. (A) Brain regional contributions for time intervals of within-session, 2-year, and 4-year intervals. Deeper colors indicate larger contributions. (B) Scatter plots demonstrate the pairwise correlation of brain region contributions across three time intervals. (C) Distribution of regional contributions (i.e., *W* coefficients) between the DMN and other brain networks, as well as between high-order cognitive networks and primary visual and sensorimotor networks. The horizontal gray lines represent the mean values for each distribution. RSNs: resting-state networks. (D) Word cloud showing -log10-transformed *p*-values of 74 brain networks from 1,000 spatial permutation tests (left), and correlations of 11 significant Neuromaps annotations.

The observed whole-brain patterns of regional contributions are highly similar across brain fingerprint extraction processes for different time intervals, showing common regions of bilateral middle temporal, superior frontal, and inferior parietal cortices (Fig. 3A and Supplementary Table S6). High correlations were observed between the contribution patterns of the within-session and the 2-year interval (*r* = 0.949, *p* < 0.001), between the within-session and the 4-year interval (*r* = 0.948, *p* < 0.001), and between the 2-year and 4-year intervals (*r* = 0.949, *p* < 0.001) (Fig. 3B). These results indicate that the contributions of brain regions to individualized functional connectivity patterns are prominent and relatively stable over time.

We then examined the spatial distributions of the observed regions in terms of brain functional networks (Yeo et al., 2011). For simplicity, we report results of the brain fingerprint extraction process for 4-year intervals, with results of within-session and 2-year intervals shown in Supplementary Tables S7 and S8. We found that regions from the default mode network (DMN) showed the highest level of the coefficient *W*, which was significantly higher than the rest of the functional networks (*t*(217) = 5.959, *p* < 0.001; Fig. 3C). The group of higher-order cognitive networks, namely the DMN, frontoparietal network (FPN), and the ventral attention network (VAN), showed significantly higher levels of *W* than primary visual and sensorimotor networks (*t*(176) = 5.618, *p* < 0.001; Fig. 3C).

The observed patterns of regional contributions were further annotated using the neuromaps (Markello et al., 2022) to provide neurobiological explanations of those key regions. First, considering the top 10 functional gradients proposed by Margulies et al. (2016), the observed pattern of coefficient *W* was significantly correlated to the principal gradient (*r* = 0.379, *p* = 0.001; false discovery rate (FDR) corrected *p* < 0.05 across 74 brain maps in the neuromaps; Fig. 3D), namely, the gradient from primary sensorimotor to higher-order association regions. Likewise, similarly significant associations were observed for the sensorimotor-association (SA) axis in the brain (*r* = 0.359, *p* = 0.001; FDR corrected) (Sydnor et al., 2021). Second, significant associations were observed for individual variability in functional brain connectivity in adults (*r* = 0.354, *p* = 0.002; FDR corrected), as reported by Mueller et al. (2013), suggestive of the consistent patterns of brain functional variability between adults and adolescents. Third, the pattern of coefficient *W* showed significant correlations with the PET tracer binding to 5-HT4 (*r* = 0.336, *p* = 0.001), 5-HT2a (*r* = 0.291, *p* = 0.003), and 5-HTT (*r* = -0.285, *p* = 0.005; FDR corrected) (Beliveau et al., 2017). Additionally, the pattern of regional contributions demonstrated a positive correlation with the evolutionary surface expansion from macaques to humans (*r* = 0.298, *p* = 0.007; FDR corrected; Fig. 3D) (Xu et al., 2020), implicating the potential association between individual variability during human brain development and human-specific configurations in brain evolution (Li et al., 2021; Mueller et al., 2013).

### 3.3 The genetic interpretation of brain fingerprints

**Fig. 4.**
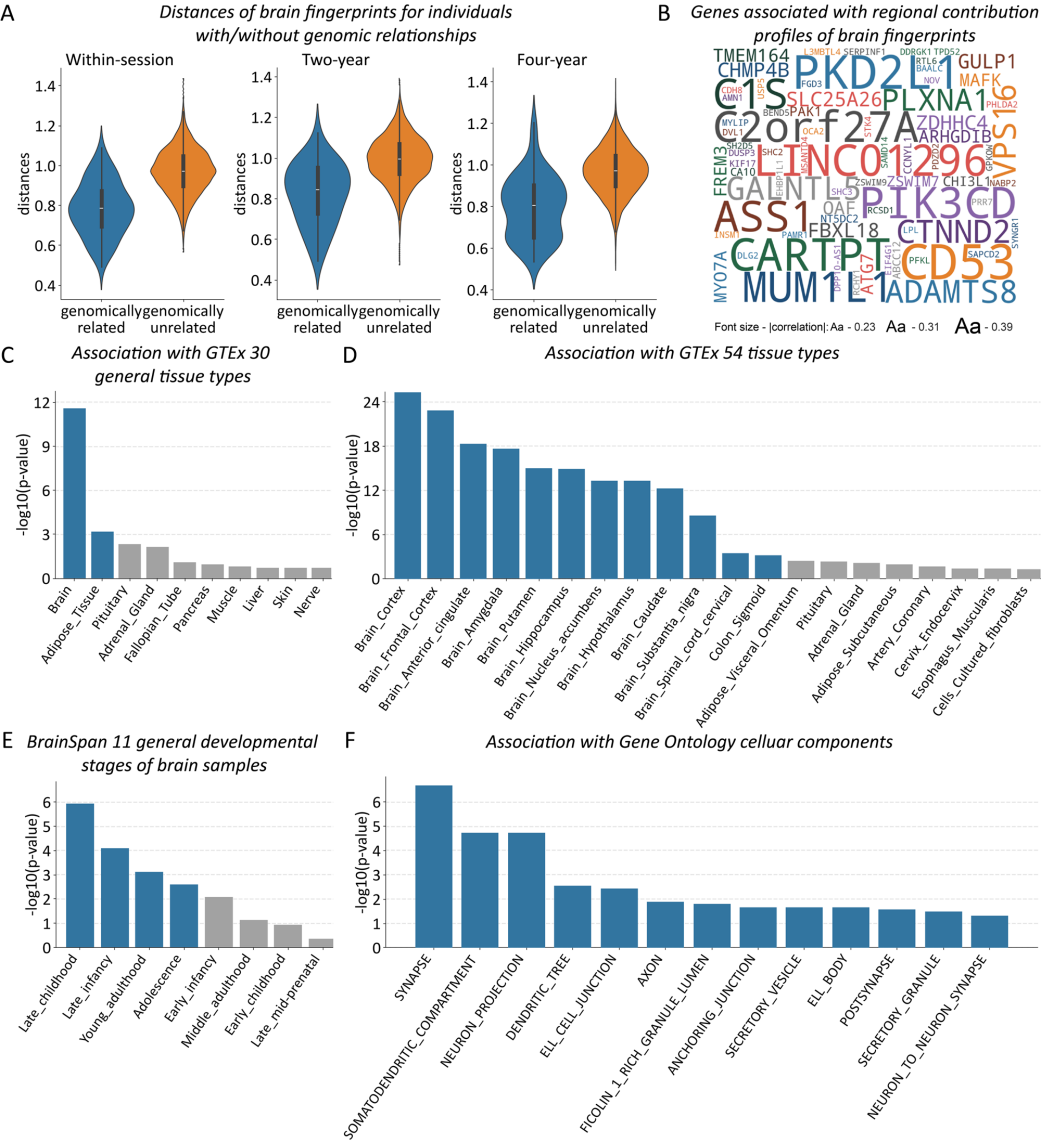
Association of brain fingerprints with genetics. (A) Brain fingerprint distances and genomic relationships. Violin plots comparing brain fingerprint distances between sample pairs with strong versus weak genomic relationships across three time intervals. White lines indicate medians; black bars show 25th to 75th percentiles. (B) Genes significantly correlated with the regional contribution patterns of brain fingerprinting. (C) Enrichment in gene sets with upregulated expressions across 30 general tissue types. (D) Enrichment in gene sets with upregulated expressions across 54 brain tissue types. (E) Enrichment in gene sets with upregulated expressions in brain tissues across different life stages. (F) Enrichment in gene sets involved in the Gene Ontology cellular components. Blue indicates significance (FDR corrected *p* < 0.05); gray indicates non-significance.

We next compared the distance of brain fingerprints between subjects with a strong genomic relationship (e.g., siblings or twins, pi_hat > 0.5) and the rest of the subjects across three time intervals. For the brain fingerprints extracted within the same session, subjects with a strong genomic relationship tended to show smaller distances of brain fingerprints than the rest of the subjects (*t*(6,318) = -12.330, *p* < 0.001; Fig. 4A). Similar results were observed for the brain fingerprints extracted across the 2-year interval (*t*(1,890) = -7.467, *p* < 0.001) and 4-year interval (*t*(3,780) = -9.101, *p* < 0.001; Fig. 4A). These results confirm that a strong genomic relationship is associated with more similar brain fingerprints at different developmental stages.

Furthermore, we identified genes related to the extracted brain fingerprints, revealing 245 genes that showed significantly positive correlations with the regional contribution profile for brain fingerprinting between baseline and 4-year data (*p* < 0.01, corrected for spatial autocorrelation, FDR corrected; Fig. 4B). The identified genes showed significant enrichment with gene sets that show upregulated expression in the brain (*p* < 0.001, FDR corrected across 30 general tissue types), in particular for gene sets with upregulated expressions in the frontal cortex (*p* < 0.001, FDR corrected across 54 tissue types; Fig. 4C, D). These genes also showed significant enrichment with gene sets that are upregulated in brain tissues from late childhood, late infancy, young adulthood, and adolescence (adjusted *p* < 0.05, FDR correction; Fig. 4E). Considering gene sets included in the gene ontology (GO), our identified genes were found to be significantly associated with cellular components of *synapses*, neuronal projections, and related structures (adjusted *p* < 0.05, FDR correction; Fig. 4F).

### 3.4 Cognitive correlates of the identified brain fingerprints

We then investigated whether brain fingerprints generated by Metric-BolT were associated with cognitive behaviors. The regression model revealed a significant association between baseline brain fingerprints and cognitive performance for fluid intelligence (Cognition Fluid Composite, *F*(936) = 1.282, *p* = 0.027), crystallized intelligence (Crystallized Composite, *F*(942) = 1.405, *p* < 0.001), and executive function assessed through cardsort (Dimensional Change Card Sort, *F*(970) = 1.290, *p* = 0.008). Results of other cognitive tests are given in Supplementary Table S9. These results demonstrate that the identified brain fingerprints are associated with individual differences in cognitive abilities.

### 3.5 Application of Metric-BolT on adult fMRI data

We additionally used the HCP data (Van Essen et al., 2013) and tested the performance of Metric-BolT in individual identification. The zero-shot transfer experiment showed a 100% SR in individual identification, while the connectome-based approach achieved 81.6% SR, suggesting the effectiveness of our brain fingerprinting method in extracting unique features in the adult brain. When the fMRI time series length was adjusted to align with the 375 time points employed in this paper, the SRs dropped to 91.5%, 86.2%, and 89.3%, for the models trained with 3 time intervals (i.e., within session, 2 years, and 4 years, respectively), while the connectome-based SR declined to 63.2%.

## 4 Discussion

Here we introduce a novel deep learning framework, Metric-BolT, which integrates metric learning techniques with transformer blocks to capture brain functional fingerprints during the development of early adolescence. This framework demonstrates high precision in individual identification using longitudinal fMRI data over intervals of up to 4 years. Brain fingerprints extracted by Metric-BolT point out the high individual variability of higher-order cognitive networks throughout development. Moreover, the brain fingerprints extracted by our framework show significant associations with cognitive abilities, including fluid intelligence, crystallized intelligence, and executive function. These brain fingerprints are also influenced by genetic factors, suggesting a hereditary component in individual brain characteristics. These findings demonstrate that brain functional fingerprints are effective and reliable for the developing brain, highlighting the uniqueness of brain functioning over the years in early adolescence.

Our results of SR within one session point out that intrinsic brain functional fingerprints become increasingly discriminative along with age, implicating that individuals progressively become unique throughout childhood and early adolescence. This is in line with previous findings showing the emergence of individual-specific brain functional organization during neurodevelopment and the growing uniqueness of brain networks with age (Kaufmann et al., 2017). This developmental trajectory prominently demonstrates that brain functional organization is a dynamic, continuous process of neural individualization, constantly adapting to unique experiences and environmental demands, and progressively forming a distinctive “brain fingerprint”. Our deep learning-based brain fingerprint identification results not only support this conclusion but also extend this by showing high precision of individual identification with a time span extending up to 4 years, indicating remarkable distinctiveness of the fingerprint over time. These findings corroborate the notion that children’s functional connectomes maintain and even enhance their self-stability throughout neurodevelopmental maturation (Graff et al., 2022).

Brain fingerprinting during development was noted to be driven by higher-order cognitive networks, in particular the DMN. These results are consistent with previous findings showing that higher-order association regions demonstrate substantial inter-subject variability and play crucial roles in coordinating large-scale brain activity (Cole et al., 2013; Power et al., 2011). Additionally, the DMN not only coordinates with other brain activities but also participates in self-awareness and self-referential processing (Molnar-Szakacs & Uddin, 2013), playing an important role in development (Supekar et al., 2010). This pattern of individual variability is known to reflect a sensorimotor-to-association axis of cortical organization (Huntenburg et al., 2018; Mueller et al., 2013), which has been suggested to arise from the temporal sequence of neurogenesis (Cahalane et al., 2012, 2014; Charvet & Finlay, 2014) and to capture the differentiated maturation rate across the cortex. Linking neurodevelopment to evolution further revealed the pattern of individual variability to be related to evolutionary cortical expansion (Hill et al., 2010; Sydnor et al., 2021), which might be coordinated by the underlying gene transcription of evolutionarily important genes like the human-accelerated regions (Li et al., 2021; Wei et al., 2019). These observations also imply that our deep-learning-based method of individual identification is biologically explainable in terms of brain development.

Our findings further demonstrate the genetic influence on brain functional fingerprinting, showing a significant difference in brain fingerprint distances between individuals with comparable and contrasting genetic backgrounds. These results align with and extend previous research, suggesting that the highly stable individual-specific characteristics may be attributed to epigenetic factors during prolonged developmental maturation (Colclough et al., 2017; Petanjek et al., 2011). Regardless of age group, genetic inheritance significantly affects the uniqueness of functional connectivity, with stronger genomic kinship leading to more similar connectivity patterns. This indicates that genetic inheritance plays a fundamental role in shaping the uniqueness of brain functional organization from early development through to adulthood (Demeter et al., 2020). The heritability of brain functional connectivity is primarily driven by higher-order association cortices, manifested in the FPN and DMN (Miranda-Dominguez et al., 2018). Our study provides novel insights into how genetic factors shape brain functional organization during adolescence. Moreover, our reported genes that correlated with regional contributions to brain fingerprinting might implicate genetic factors that are associated with normal or abnormal neurodevelopment. For example, abnormal PIK3CD has been noted in diverse neurodevelopmental disorders, including schizophrenia, autism, and developmental delay (Hood et al., 2019). Future studies could investigate how these genetic signatures serve as biomarkers for atypical development.

The performance dropped when shortening the fMRI time series from 1,200 to 375 points. This is primarily attributed to short sequences acting as low signal-to-noise ratio (SNR) “dynamic snapshots” that cannot comprehensively represent an individual’s stable and unique neural characteristics, thus limiting the model’s identification performance to the information ceiling of that data length. To mitigate this issue and improve the model’s generalization ability, a promising strategy is to adopt a multi-scale training paradigm: specifically, pre-training the model on a dataset containing sequences of various lengths to force it to learn a more robust feature extraction method that is independent of specific scan durations.

Several considerations should be taken into account when interpreting our findings. First, as noted in the Introduction, brain fingerprinting techniques face challenges when scaled to massive cohorts (e.g., *N* > 10,000). While our sample size is substantial for a neuroimaging study, and the five-fold cross-validation results (e.g., 86.1% average SR over 4 years) confirm the robustness of the Metric-BolT model, studies with larger samples are still warranted to test the scalability and generalizability of our model. Secondly, individual identification in this study relies on resting-state fMRI data. Future work could extend brain fingerprinting to task-based fMRI data or incorporate other imaging modalities (such as EEG, Structural MRI, and diffusion MRI), particularly in identifying individuals across different tasks (Abbas et al., 2020). Third, unlike previous approaches using FC (Chiêm et al., 2022), the Metric-BolT model is built on time series data. While this might overlook certain information traditionally captured by FC analysis, our results suggest that this deep learning model can effectively capture complex brain dynamics (Cai et al., 2018; Peng et al., 2023) that might be missed by conventional FC-based methods. Thus, combining dynamic FC and static FC might be a promising direction to provide more comprehensive insights into studying individual differences (Griffa et al., 2022). Finally, exploring the stability of the brain from adolescence through adulthood is an interesting direction, as our understanding in this area remains limited. Studying the developmental trajectory of functional connectivity across these life stages can help us better understand the long-term changes and maturation of individual neural networks from youth to adulthood.

To summarize, we proposed a distance-based deep metric learning framework to provide an intuitive and interpretable approach to extract fingerprints and dissect inter-individual variability in functional dynamics in early adolescents. This method achieved a high precision in individual identification over as long as 4 years and pointed to the contribution of higher-order association networks in fingerprinting. The extracted brain fingerprint exhibits the association with behavior and is modulated by genetic factors. Our proposed approach provides new insights into the unique brain developmental trajectory in early adolescents.

## Supplementary Material

Supplementary Material

## Data Availability

The raw MRI data were obtained through NIMH Data Archive (https://nda.nih.gov/) and connectomeDB (https://www.humanconnectome.org/). The codes of this study are given at https://github.com/CAIMI-WEIGroup/MetricBolT.
