# Supplementary material for: Functional fingerprinting for the developing brain using deep metric learning
Source: Imaging Neurosci (Camb). 2026 Jan 27;4:IMAG.a.1112. doi: 10.1162/IMAG.a.1112 (PMC12849230; doi:10.1162/IMAG.a.1112)
Supplement: Supplementary Material [file IMAG.a.1112_supp.pdf]

Supplementary Methods

Metric-BolT implementation

For encoding time series into feature vectors (i.e., brain fingerprint), we employed the Blood-oxygen-level-dependent Transformer (BolT) model [28] to optimally represent the fMRI data from the ABCD dataset in the embedding space. Based on the transformer architecture, each time point's BOLD response in the time series is treated as a BOLD token, and BolT can directly operate on these BOLD tokens. To capture local representations, the BolT divides the time series into overlapping temporal windows and employs a series of transformer blocks. This structure allows for the encoding of window-specific representations of BOLD tokens. BolT incorporates a novel fused window attention mechanism, which leverages cross attention and token fusion among overlapping windows, enhancing expressiveness across broad time scales without increasing computational complexity. The cross attention component facilitates interactions between base BOLD tokens in a given window and fringe tokens in neighboring windows before encoding. Specifically, the Query (Q) vectors are generated only from the local tokens within a specific window (the 'base' tokens). However, the Key (K) and Value (V) vectors, which form the searchable information pool, are generated from a wider receptive field that includes both the base tokens and the adjacent 'fringe' tokens from neighboring windows. This forces the local Queries to probe the expanded Key/Value space, thereby allowing the base tokens to selectively integrate information from outside their immediate window. This process infuses each local representation with crucial context from its direct temporal neighbors before the main encoding step. The model's use of overlapping windows means that a single token is processed within multiple, distinct local contexts. As a result, after the attention calculation, several different latent representations are generated for the same token, each contextualized by a different window. Specifically, the vector representations for every window of a particular token are summed together, and this sum is then divided by the number of windows in which the token appeared. This resulting averaged vector serves as the final, 'fused' representation of that token for subsequent processing layers. To achieve a hierarchical transition from local to global representations, the degree of window overlap in transformer blocks

gradually increases. Window-specific CLS tokens are introduced to maintain local sensitivity and compatibility with the hierarchical model structure. The final step involves averaging the encoded CLS tokens across windows. This consolidation process integrates local and global information, resulting in a highly representative encoding of the original time series.

The Metric-BolT model is designed as a deep Transformer architecture comprising a cascade of four blocks. Its hidden dimensionality is set to 219, precisely matching the number of brain regions in the input data. Each block consists of a Fused Window Multi-Head Self-Attention (FW-MSA) module and a feed-forward network (MLP), and incorporates Layer Normalization, skip connections, and a dropout rate of 0.1.

The model processes the time series using windows with a base size of 25 and a stride of 10. Its receptive field dynamically expands across the layers; the contextual fringe region progressively grows from 0 to 90 tokens, enabling a smooth transition from local to global representations. The attention mechanism employs 36 parallel attention heads, each with a dimensionality of 20. The concatenated 720-dimensional output from these heads is subsequently mapped back to the model's required 219-dimensional hidden state via a linear projection for further processing.

To compare feature vector pairs for distance, we focused on two critical components: the distance measure and the loss function. Given that our Metric-BolT model maps the original input into a high-dimensional vector space, we use cosine similarity to measure the similarity between two vectors and use 1 minus cosine similarity as the distance measure. Cosine similarity is computationally efficient in high-dimensional spaces, with values ranging from -1 to 1, where higher values indicate greater similarity between vectors. For the loss function, we employed TripletMarginLoss [69], which outperformed alternatives such as MultiSimilarityLoss and CircleLoss in terms of efficiency and effectiveness in our experiment. The TripletMarginLoss is defined as:

$$\mathrm{L}(a,p,n)=\max\{\mathrm{d}(a,p)-\mathrm{d}(a,n)+margin,0\} \tag{1}$$

L represents the triplet loss function, where a, p and n denote the anchor, positive sample (same class as a), and negative sample (different class from a), respectively, as illustrated in Fig 1.

Evaluation metrics

The success rate was defined as the proportion of subjects whose identity was accurately identified:

$$SR = \frac{\text{number of correctly identified subjects}}{\text{number of total subjects}} \quad (2)$$

Correct identification was scored as 1 point for the correct match (the identified identity matches the true identity) and 0 points for an incorrect match. Given that our metric learning shares conceptual similarities with clustering, we employed two additional clustering metrics to provide a more comprehensive analysis of the individual identification performance. For this, each subject was treated as a class, with their corresponding feature vectors serving as samples. The intra-class to inter-class distance ratio was utilized to assess the compactness within classes relative to the separation between classes. The ratio is calculated as:

$$CR = \frac{\text{average intra-class distance}}{\text{average inter-class distance}} \quad (3)$$

where the intra-class distance is the average distance between samples within the same class, while the inter-class distance is the average distance between samples from different classes. The CR ranges from 0 to 1, with smaller values indicating that samples within a class are more compact and better separated from those in other classes. Complementing this, the silhouette coefficient (SC) was employed to quantify the cohesion and separation of the resulting clusters. The silhouette coefficient for a sample i is defined as:

$$SC(i) = \frac{b(i) - a(i)}{\max\{a(i), b(i)\}} \quad (4)$$

$$SC = \frac{1}{N}\sum_{i=1}^{N} SC(i) \quad (5)$$

where $a(i)$ is the mean distance between i and all other vectors in the same subject, and $b(i)$ is the mean distance between i and all vectors in the nearest neighboring subject. The overall silhouette coefficient is the average across all subjects and ranges from -1 to 1, where values near 1 indicate that the vector is well-matched to its own subject and poorly-matched to neighboring subjects. Values around 0 suggest the

vector lies near the boundary between two subjects. Negative values indicate that the vector might have been misclassified. This comprehensive evaluation framework allows for a rigorous assessment of the method's performance from multiple perspectives, enhancing the robustness of our findings.

Model interpretations using the transformer block

For each transformer block, we first compute the attention map for each window:

$$\overline{A_{mi}} = E_h((A_i)^+) \tag{6}$$

where m denotes the index of the transformer block, i denotes the index of the window, E_h denotes the averaging operator across attention heads for aggregation, + denotes rectification to prevent negative values, and then aggregate the attention matrix for all time windows for each transformer block to form a global attention graph $\overline{A_G}$, ($\overline{A_G} \in R^{(F+T)*(F+T)}$), where F denotes the number of time windows and T denotes the number of BOLD tokens). The values in $\overline{A_G}$ represent the attention between each token. Specifically, the $\overline{A_G}[:F, F:]$ represents the attention weights from the CLS tokens to the BOLD tokens. This can be interpreted as the contribution or influence of the BOLD tokens on the CLS tokens.

Next, a token-relevance matrix $Rel[0]$ is initialized to represent the interactions between each token in the whole procedure of extracting brain fingerprints, which is initialized as an identity matrix of size $(F+T)*(F+T)$, indicating that each token has only autocorrelation, and then the token correlation maps of each transformer block are updated step by step using the attention matrix:

$$Rel[m+1] = Rel[m] + \overline{A_G} Rel[m] \tag{7}$$

Following the calculation of the token-relevancy map, importance weights for input BOLD tokens are finally derived as:

$$w_{imp} = \frac{1}{F} \sum_{i=0}^{F-1} Rel[M](i, F:) \tag{8}$$

The final importance weight of each BOLD token was derived from its cross-window average correlation with the CLS token, providing a measure of its overall contribution to the brain fingerprint.

Site-conserved nested five-fold cross-validation

To ensure a robust and unbiased evaluation of model performance, we employed a site-conserved nested 5-fold cross-validation procedure. The dataset was first partitioned into five non-overlapping outer folds, ensuring that subjects from the same scanning site were exclusively grouped into a single fold (site-conserved). The model was then iteratively trained and tested five times (outer loop); in each iteration, a different outer fold served as the test set, and the remaining four were designated as the training set. Crucially, the training set was further split into inner folds for hyperparameter optimization and validation, utilizing the optimal margin for training (Supplementary Table S1). The final performance metrics, reported in Supplementary Table S4, represent the average performance across all five outer folds, providing a comprehensive and reliable estimate of the model's generalizability. By employing this method, the nested cross-validation ensures that hyperparameter selection is independent of the final test set, thus offering a more rigorous evaluation of the model.

Analysis on a consistent cohort of subjects

We identified the list of subjects present in the dataset for each time span and then took the intersection of these lists. This step ensured that our final analysis dataset exclusively contained subjects who provided data at all observed time intervals. Subsequently, this standardized dataset, comprised of the common cohort, was partitioned a single time into training, validation, and test sets using an 8:1:1 ratio. Critically, this exact same partition—meaning the same subjects were allocated to the training, validation, and test sets respectively—was used for all experiments across the different time spans. This methodology completely eliminates potential confounding variables arising from subject variability or random data splits, thereby guaranteeing that any observed differences in model performance can be directly and reliably attributed to the core experimental variable: the time span.

Inclusion of deep learning baselines for comparative analysis

To provide a more comprehensive and rigorous evaluation of our proposed

Transformer-based model, we recognized that a comparison solely against the traditional connectome fingerprinting method based on Pearson correlation was insufficient. Therefore, we expanded our comparative analysis to include two contemporary deep learning-based baseline methods from recent literature. This allows for a more robust benchmarking of our model's performance against the state-of-the-art. The selected baseline models are 1) An autoencoder with sparse dictionary learning, as proposed by Cai et al. (2021) and 2) A conditional Variational Autoencoder (VAE), as proposed by Lu et al. (2024). To ensure a fair and direct comparison, both baseline models were trained and evaluated on the same standardized dataset and under the same experimental protocols as our model. The detailed comparative performance results are presented in Supplementary Table S5.

Genetic data analysis

In the process of genotype data quality control, we first calculate the missing rates for samples and Single Nucleotide Polymorphisms (SNPs) to exclude low-quality data. Next, we calculate the minor allele frequency (MAF), which helps in categorizing and analyzing the frequency of variants. Then, through the Hardy-Weinberg equilibrium exact test, we can detect potential genotyping errors. To ensure data accuracy, we apply multiple filters to exclude samples and SNPs that do not meet standards. Meanwhile, through linkage disequilibrium (LD) pruning, we effectively remove SNPs with strong linkage disequilibrium, reducing redundancy in the analysis. Additionally, we check the heterozygosity coefficient of samples, using the inbreeding coefficient to identify and exclude potentially contaminated samples. Finally, we select specific samples and SNPs for further analysis to enhance the reliability and accuracy of the data.

By calculating IBD (the proportion of shared alleles) and evaluating pi_hat (the kinship coefficient), we assess the genetic relationships between individuals. IBD refers to the situation where two individuals share identical alleles and these alleles are inherited from a common ancestor rather than arising from mutation or other means. pi_hat is a statistical measure used to quantify the concept of IBD, calculated using the following formula:

$$pi\_hat = p(IBD = 2) + 0.5 * p(IBD = 1) \tag{9}$$

where $p(IBD = 2)$ represents the probability that both alleles come from a common ancestor, while $p(IBD = 1)$ signifies the probability that only one allele comes from a common ancestor. The value of pi_hat ranges from 0 to 1, reflecting the extent to which two individuals share alleles.

Correlation analysis between brain fingerprints and cognitive abilities

When performing the regression analysis to link brain fingerprint features with cognitive scores, we included each subject's age as a covariate. By modeling the effects of both age and brain fingerprint features simultaneously, this approach allows us to statistically isolate the unique variance in cognitive scores explained by the brain fingerprint, independent of shared age-related developmental trends. Our main findings remained significant after applying this statistical control, confirming the robustness of the observed brain-cognition relationship. The detailed results of this analysis, including the R-squared values, are presented in Supplementary Table S10.

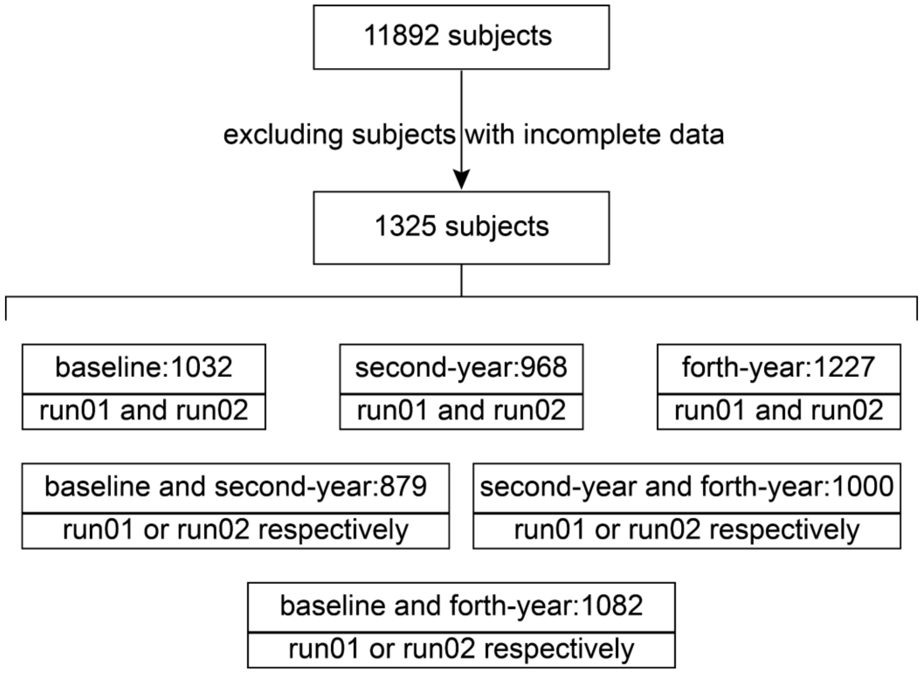

Fig S1. Participant Screening Pipeline. Subjects with incomplete data were defined as those without the fourth-year follow-up data or those lacking either T1 imaging or resting-state fMRI data. "run01 and run02" denotes the availability of both runs at the specified time point. "run01 or run02 respectively" signifies that at each of two distinct time points, at least one of the two runs (run01 or run02) is available.

Supplementary Tables

Table S1. Optimized margins in nested cross-validation

fold	within-session	two-year	four-year
fold 1	0.71	0.71	0.68
fold 2	0.72	0.67	0.66
fold 3	0.71	0.72	0.73
fold 4	0.73	0.73	0.71
fold 5	0.65	0.66	0.67

Table S2. Final hyperparameter configuration

Parameters	Value	Source
margin	0.7	Tuned
batch size	8	Fixed
windowSize	25	Fixed
shiftCoeff	0.4	Fixed
fringeCoeff	2	Fixed
dim	219	Fixed
numHeads	36	Fixed
lr	2e-4	Inherited from BolT
minLr	2e-5	Inherited from BolT
maxLr	4e-4	Inherited from BolT
nOfLayers	4	Inherited from BolT
headDim	20	Inherited from BolT
focalRule	expand	Inherited from BolT
mlpRatio	1.0	Inherited from BolT
attentionBias	True	Inherited from BolT
drop	0.1	Inherited from BolT
attnDrop	0.1	Inherited from BolT
lambdaCons	1	Inherited from BolT

Table S3. Training and testing sample sizes for each fold in site-conserved cross-validation

fold	within-session		two-year		four-year	
	train	test	train	test	train	test
fold 1	983	244	790	201	851	210
fold 2	978	249	796	195	847	214
fold 3	982	245	793	198	843	218
fold 4	981	246	793	198	852	209
fold 5	984	243	792	199	851	210

Table S4. The SRs of site-conserved five-fold cross-validation

fold	within-session	two-year	four-year
fold 1	97.5%	90.1%	92.3%
fold 2	96.4%	90.8%	83.6%
fold 3	98.0%	86.9%	90.8%
fold 4	97.6%	93.4%	80.0%
fold 5	96.3%	90.0%	83.8%
Average SR	97.1%	90.2%	86.1%

Table S5. The SRs of comparative analysis

Method	within-session	two-year	four-year
AE (Cai et al., 2021)	76%	78%	83%
VAE (Lu et al., 2024)	90%	80%	84%
Ours	97%	93%	87%

Table S6. The top five brain regions contributing to brain fingerprinting across three time spans

within-session		two-year		four-year	
Region	*W* coff	Region	*W* coff	Region	*W* coff
lh-middletemporal_3	0.041	lh-middletemporal_3	0.052	lh-middletemporal_3	0.043
lh-superiorfrontal_3	0.035	lh-supramarginal_3	0.036	lh-superiorfrontal_3	0.039
lh-inferiorparietal_3	0.034	lh-superiorfrontal_3	0.033	lh-middletemporal_2	0.032
lh-middletemporal_2	0.028	lh-middletemporal_2	0.032	lh-inferiorparietal_5	0.029
lh-supramarginal_3	0.025	lh-inferiorparietal_3	0.031	rh-supramarginal_3	0.025

Table S7. Correlations of brain network *W*-values across within-session and two-year intervals

Brain map	within-session		two-year	
	r	*p*-value	*r*	*p*-value
fcgradient01	0.379	0.001	0.343	0.001
5-HT4	0.336	0.001	0.347	0.001
SAaxis	0.360	0.001	0.325	0.002
intersubjvar	0.355	0.002	0.316	0.002
5-HT2a	0.291	0.003	0.300	0.003
mamdam	-0.173	0.004	-0.183	0.004
fmpepd2	0.281	0.005	0.228	0.005
omar	0.260	0.005	0.237	0.005
dasb	-0.264	0.005	-0.240	0.005
evoexp	0.298	0.007	0.295	0.007

Table S8. Regional contributions of each brain network versus the rest of the brain across within-session and two-year intervals

Networks	within-session		two-year	
	t-value	*p*-value	*t*-value	*p*-value
Visual Network	-3.076	0.002	-2.726	0.007
Sensorimotor Network	-3.042	0.003	-2.647	0.009
Dorsal Attention Network	-0.557	0.578	-0.320	0.749
Ventral Attention Network	1.688	0.093	1.523	0.129
Limbic Network	-2.226	0.027	-1.751	0.081
Fronto-parietal Network	0.334	0.739	-0.624	0.533
Default Mode Network	6.021	<0.001	5.517	<0.001

Table S9. Associations between brain fingerprints and cognitive behaviors obtained from NIH toolbox

Cognitive score	*F*-value	*p*-value
Cognition Fluid Composite	1.228	0.027
Crystallized Composite	1.521	<0.001
Dimensional Change Card Sort	1.290	0.008
Cognition Total Composite	1.503	<0.001
Oral Reading Recognition	1.242	0.020
Picture Vocabulary	1.469	<0.001
List Sorting Working Memory Test	1.194	0.047
Flanker Inhibitory Control and Attention Test	1.109	0.164
Pattern Comparison Processing Speed Test	1.101	0.181
Picture Sequence Memory Test	0.930	0.741

Table S10. Cognitive correlates of brain fingerprints independent of age effects

Cognitive score	*F*-value	*p*-value	*R*-squared
Cognition Fluid Composite	1.228	0.026	0.274
Crystallized Composite	1.517	<0.001	0.317
Dimensional Change Card Sort	1.284	0.008	0.274
Cognition Total Composite	1.495	<0.001	0.315
Oral Reading Recognition	1.238	0.021	0.267
Picture Vocabulary	1.477	<0.001	0.303
